# Human Malignant Rhabdoid Tumor Antigens as Biomarkers and Potential Therapeutic Targets

**DOI:** 10.3390/cancers14153685

**Published:** 2022-07-28

**Authors:** Timothy Hua, Ziwei Zeng, Junji Chen, Yu Xue, Yan Li, Qingxiang Sang

**Affiliations:** 1 Department of Chemistry and Biochemistry, Florida State University, Tallahassee, FL 32306-4390, USA; tph16c@fsu.edu (T.H.); zengzw@mail2.sysu.edu.cn (Z.Z.); chenjj47@mail2.sysu.edu.cn (J.C.); yx21@fsu.edu (Y.X.); 2 Department of Chemical and Biomedical Engineering, FAMU-FSU College of Engineering, Florida State University, Tallahassee, FL 32310-6046, USA; yli4@fsu.edu; 3 Institute of Molecular Biophysics, Florida State University, Tallahassee, FL 32306-4380, USA

**Keywords:** malignant rhabdoid tumor antigens, atypical teratoid rhabdoid tumor (ATRT), immunotherapy, cancer biomarkers, mesothelin (MSLN), osteopontin (OPN), matrix metalloproteinases (MMPs), therapeutic targets, cancer vaccines, mucin-16 (MUC16/CA125), alpha-fetoprotein (AFP)

## Abstract

**Simple Summary:**

Atypical teratoid rhabdoid tumor (ATRT) is a deadly type of human pediatric brain cancer without effective treatments. ATRT is mainly linked to the inactivation of a tumor suppressor gene, SMARCB1; however, additional biomarkers remain to be identified to develop novel therapeutic strategies. Therefore, different tumor antigens and extracellular matrix modulators were investigated in two human ATRT and one kidney malignant rhabdoid tumor cell lines and compared with the nonmalignant HEK293 cell line. Alpha-fetoprotein (AFP), mucin-16 (MUC16 or cancer antigen 125/CA125), osteopontin (OPN), and mesothelin (MSLN) are highly expressed in these human malignant rhabdoid cancer cell lines. Inhibiting MMPs using a small-molecule inhibitor decreased cell survival. This biomarker discovery process may lead to the identification of novel diagnostic and therapeutic strategies, such as the development of targeted and immunotherapies against cancer biomarkers, to treat cancer patients.

**Abstract:**

Introduction: Atypical teratoid rhabdoid tumor (ATRT) is a lethal type of malignant rhabdoid tumor in the brain, seen mostly in children under two years old. ATRT is mainly linked to the biallelic inactivation of the SMARCB1 gene. To understand the deadly characteristics of ATRT and develop novel diagnostic and immunotherapy strategies for the treatment of ATRT, this study investigated tumor antigens, such as alpha-fetoprotein (AFP), mucin-16 (MUC16/CA125), and osteopontin (OPN), and extracellular matrix modulators, such as matrix metalloproteinases (MMPs), in different human malignant rhabdoid tumor cell lines. In addition, the roles of MMPs were also examined. Materials and methods: Five human cell lines were chosen for this study, including two ATRT cell lines, CHLA-02-ATRT and CHLA-05-ATRT; a kidney malignant rhabdoid tumor cell line, G401; and two control cell lines, human embryonic kidney HEK293 and HEK293T. Both ATRT cell lines were treated with a broad-spectrum MMP inhibitor, GM6001, to investigate the effect of MMPs on cell proliferation, viability, and expression of tumor antigens and biomarkers. Gene expression was examined using a reverse transcription polymerase chain reaction (RT-PCR), and protein expression was characterized by immunocytochemistry and flow cytometry. Results: All the rhabdoid tumor cell lines tested had high gene expression levels of MUC16, OPN, AFP, and MSLN. Low expression levels of neuron-specific enolase (ENO2) by the two ATRT cell lines demonstrated their lack of neuronal genotype. Membrane-type 1 matrix metalloproteinase (MT1-MMP/MMP-14) and tissue inhibitor of metalloproteinases-2 (TIMP-2) were highly expressed in these malignant rhabdoid tumor cells, indicating their invasive phenotypes. GM6001 significantly decreased ATRT cell proliferation and the gene expression of MSLN, OPN, and several mesenchymal markers, suggesting that inhibition of MMPs may reduce the aggressiveness of rhabdoid cancer cells. Conclusion: The results obtained from this study may advance our knowledge of the molecular landscapes of human malignant rhabdoid tumors and their biomarkers for effective diagnosis and treatment. This work analyzed the expression of human malignant rhabdoid tumor antigens that may serve as biomarkers for the development of novel therapeutic strategies, such as cancer vaccines and targeted and immunotherapies targeting osteopontin and mesothelin, for the treatment of patients with ATRT and other malignant rhabdoid tumors.

## 1. Introduction

Malignant rhabdoid tumors are aggressive tumors that mostly appear in children under two years of age [1]. Atypical teratoid rhabdoid tumors (ATRTs) are malignant rhabdoid tumors in the brain [1,2]. ATRT is a rare disease and accounts for 2% of pediatric brain tumors and 4.4% of central nervous system tumors in children younger than five years old [3,4]. ATRT is difficult to diagnose and a deadly disease with no effective treatment. Currently, the standard treatment of ATRTs includes surgery to remove the tumor, intensive radiation, and chemotherapy. ATRT prognosis is very poor. The three-year overall survival rate is 22%, and the tumor-free survival rate is 13% [1,5,6].

ATRT has several histological features, including eosinophilic cytoplasm, large nucleoli, and filamentous cytoplasmic inclusions [7]; however, tumors may present a host of neural, epithelial, mesenchymal, or ependymal patterns that make them variable and difficult to diagnose by histology [7]. In 1999, ATRT was mainly linked to the hSNF5/INI-1/SMARCB1 gene’s inactivation on chromosome 22 [8]. As antibodies against SMARCB1 developed, immunohistochemistry effectively determined the lack of protein expression in cancerous tissue samples [9]. In addition, the unexpressed SMARCB1 protein is exclusive to ATRT and other cancers, such as kidney RT, chordoma, epithelioid sarcoma, and medullary renal cell carcinoma [10,11,12,13]. These findings suggest that SMARCB1 expression alone is neither sufficiently sensitive nor specific for ATRT. Therefore, additional biomarkers should be investigated for ATRT characterization.

CD44 is expressed on the surface of ATRT cells and tumor mesenchymal stromal cells from the rhabdoid tumor microenvironment [14]. In addition, CD44 is regulated by the BRG-1 subunit of the SWI–SNF complex [15]; hence, it was investigated in the study. The level of CD44 cleavage is significantly associated with OPN and OPN–CD44 interaction [16], suggesting that both OPN and CD44 could be potential tumor markers for ATRT. CD99 functions in monocyte migration and in endothelial cells under normal physiological conditions [17]; however, it is expressed in most ATRT cases and has been suggested to be an ATRT biomarker [18,19,20,21]. CD99 positivity has been used to eliminate tumor diagnosis as primitive neuroectodermal tumor and rhabdoid meningioma [19]. Although the chorionic gonadotropin subunit beta 3 (CGB3) gene is overexpressed in fetal cells and various cancer types [22], the connection between CGB3 and rhabdoid tumors has yet to be investigated. Alpha-fetoprotein (AFP) is essential to embryonic development because it involves crosstalk with albumin and directs specific brain region development through the neurotrophic effects of oleic acid [23]. It is also a biomarker for several cancer types, especially hepatocellular carcinoma [24,25,26]. Nucleophosmin 1 (NPM1) is important for various cellular pathways, including mRNA transport, chromatin remodeling, apoptosis, and genome stability [27]. One study showed that NPM1 is one of the most upregulated genes in ATRT, and inhibiting NPM1 could arrest cancerous cells at the G1 checkpoint [28]. Therefore, these common cancer biomarkers were chosen for this study.

Compared with medulloblastoma, ATRT has a stemness-like feature and expresses an embryonic stem cell gene profile of OCT4, NANOG, SOX2, and c-MYC [29]. Hence, embryonic markers were also investigated. In addition, SSEA1 (Stage-Specific Embryonic Antigen 1, fucosyltransferase 4, CD15), a glioblastoma tumor stem cell marker, was investigated [30]. MUC16, also called cancer antigen 125 (CA125) [31], is expressed in various types of brain cancer, such as glioblastoma [32] and medulloblastoma [33], and nonbrain cancer, such as ovarian cancer, breast cancer, pancreatic cancer, gastric cancer, and lung cancer [34,35,36,37,38]. MUC16 can bind to membrane proteins of different tissues, promote metastasis in various cancer types, induce cell proliferation and metastasis, and protect tumor cells from chemotherapy [31,35,39,40,41,42,43,44,45]. MSLN is a 40 kDa glycosyl-phosphatidylinositol (GPI)-anchored membrane protein. The normal physiological function of MSLN is not clear, but it is highly expressed in the lung, heart, spleen, liver, kidney, and testis of adult mouse tissues [46]. The protein, however, is not essential for mouse growth and reproduction [46]. MSLN might be involved in cell adhesion [47]. MSLN is overexpressed in various cancer types, including mesothelioma [47], lung cancer [48], pancreatic cancer [49,50], ovarian cancer [47,51], breast cancers [52], and meningiomas [53]. Overexpression of MSLN indicates a poor prognosis for cancer patients [48,54]. Therefore, both MSLN and MUC16 were examined to find out whether they are also ATRT biomarkers.

Matrix metalloproteinases (MMPs) are zinc-dependent endopeptidases/proteinases with the function of degrading extracellular matrix (ECM) proteins, including basement membrane and interstitial matrix [55]. Their roles in an inflammatory response and vascular diseases have been discussed [56]. MMPs are potential biomarkers of invasive and metastatic cancer cells. Our study has detected elevated expression levels of several MMPs by ATRT cells.

The expression levels of tissue inhibitors of metalloproteinases (TIMPs) and disintegrin and metalloproteinases (ADAMs) were also measured in the five cell lines. ADAMs are anchored on the cell membrane, functioning in processes of fertilization, restenosis, neurogenesis, and tumor invasion and metastasis by degrading ECM [57,58], whereas TIMPs usually serve as tentative agents targeting MMPs to treat cancer, cardiovascular diseases, multiple sclerosis, and other diseases [59]. We have demonstrated that TIMPs and ADAMs are differentially expressed by the two ATRT cell lines studied.

Diagnostic and therapeutic biomarkers with improved sensitivity and specificity are needed for ATRT due to its complexity [60,61]. ATRT biomarker discovery is an under-explored area of research. Therefore, the gene and protein expression levels of cancer biomarkers and extracellular matrix modifiers (MMPs, TIMPs, and ADAMs) were characterized in different malignant rhabdoid tumor cell lines to identify ATRT biomarkers. This study is a discovery science project that aimed to identify biomarkers for malignant rhabdoid tumor diagnosis and treatment.

## 2. Materials and Methods

### 2.1. Culturing Human Malignant Rhabdoid Tumor Cells and Nonmalignant Cells

Both CHLA-02-ATRT (ATCC^®^ CRL-3020^TM^, ATCC) and CHLA-05-ATRT (ATCC^®^ CRL-3037^TM^, ATCC) cells were cultured based on the manufacturer’s protocol. Briefly, the cells were plated in suspension using Gibco Dulbecco’s Modified Eagle Medium: Nutrient Mixture F-12 (DMEM/F12, Thermo Fisher Scientific, Waltham, MA, USA) supplemented with 2% B-27, 20 ng/mL of epidermal growth factor (EGF, 78,006.1, STEMCELL Technologies, Vancouver, BC, Canada), and 20 ng/mL of fibroblast growth factor (FGF)-2 (78,003.1, STEMCELL Technologies), in a non-treated 6-well plate at 37 °C, 5% CO_2_. The initial cell density was 1 × 10^6^ cells/mL. The cell density was determined every 2 to 3 days using a hemocytometer (see Section 2.2), and new media was added accordingly. When the cells reached high confluence (2 × 10^6^ cells/mL), they were passaged at the ratio of 1:6 into a new vessel.

HEK293 (ATCC^®^ CRL-1573^TM^, ATCC) and HEK293T (ATCC^®^ CRL-11268^TM^, ATCC) cells were cultured in DMEM supplemented with 10% fetal bovine serum (FBS) at 37 °C at 5% CO_2_. The cells were passaged when they reached 80% confluence using trypsin (37 °C, 5% CO_2_, 5 min). G401 (ATCC^®^ CRL-1441^TM^, ATCC) was cultured based on the ATCC protocol. Briefly, the cells were cultured in McCoy’s 5A Medium (ATCC^®^ 30-2007^TM^, ATCC) supplemented with 10% FBS. The cells were also passaged upon reaching 80% confluence.

### 2.2. Cell Proliferation Assay and Imaging

Both CHLA-02 and CHLA-05 were harvested from suspension and washed three times with PBS. Then, the samples were treated with Accumax (AM105, Innovative Cell Technologies, Inc., San Diego, CA, USA) for 20 min at 37 °C at 5% CO_2_. Then, 6% FBS in phosphate-buffered saline (PBS) was used to quench the reaction. A mixture of the cell solution with Trypan Blue 0.4% (T10282, Thermo Fisher Scientific) at a 1:1 ratio was pipetted onto Countess chamber slides (C10228, Thermo Fisher Scientific) using the Countess^®^ II FL instrument (AMQAF1000, Thermo Fisher Scientific). The total cell number and cell viability were reported.

The images of CHLA-02 and CHLA-05 aggregates were taken using an Olympus IX70 microscope (Melville, NY). In addition, some of the aggregates of CHLA-02 and CHLA-05 were replated on *Matrigel*^TM^-coated surfaces to let the cells spread outward from the spheroids. The images of these cells were also taken using an Olympus IX70 microscope.

### 2.3. Reverse Transcription Polymerase Chain Reaction (RT-PCR) Analysis

An E.Z.N.A.^®^ Total RNA Kit I (R6834, OMEGA Bio-Tek, Norcross, GA, USA) was used to isolate the total RNA from each cell line, and an RNA Clean & Concentrator-5 kit (R1013, Zymo Research, Irvine, CA, USA) was used to clean and concentrate the total mRNA samples. The reverse transcription required 2 μg of the total RNA, anchored oligo-dT primers (Operon, Huntsville, AL, USA), and Superscript III (Invitrogen, Carlsbad, CA, USA) and was performed according to the manufacturer’s protocol. The primers specific to target genes (Tables S1, S5, and S7) were designed using Primer-BLAST (NCBI), and the melting temperature was checked using NetPrimer Analysis (PREMIER Biosoft, Palo Alto, CA, USA). The β-ACTIN (*ACTB*) gene was used as an endogenous control for normalizing expression levels. RT-PCR was performed on an ABI7500 instrument (Applied Biosystems, Waltham, MA, USA) using SYBR1 Green PCR Master Mix (Applied Biosystems). The amplification reactions were 2 min at 50 °C, 10 min at 95 °C, and 40 cycles of 95 °C for 15 s, 55 °C for 30 s, and 68 °C for 30 s. The fold variation in gene expression was quantified using the comparative 2^−ΔΔCT^ method based on the comparison of expression of the target genes (normalized to the endogenous control β-actin) among different conditions.

### 2.4. Immunocytochemistry

The cells were replated on a *Matrigel*^TM^-coated 96-well plate and then fixed with 4% paraformaldehyde (PFA). For nuclear markers, the cells were permeabilized with 0.5% Triton X-100. The samples were then blocked in 2% FBS in phosphate-buffered saline (PBS) for 1 h and incubated with various mouse antibodies in a blocking buffer at 4 µg/mL (Table S3) overnight at 4 °C. After washing with PBS, the cells were incubated with the corresponding secondary antibody for one hour at room temperature (Table S4). Finally, the samples were stained with Hoechst 33342 and visualized using a fluorescent microscope (Olympus IX70, Melville, NY, USA).

### 2.5. Flow Cytometry

Briefly, 1 × 10^6^ cells/sample were fixed with 4% PFA and permeabilized with cold methanol. The samples were then blocked with 2% FBS in PBS and stained with the corresponding markers in 2% FBS in PBS overnight at 4 °C. The secondary Alexa Fluor 488 or 594 was used and incubated for an hour at room temperature. The samples were washed with PBS before being acquired with a BD FACSCanto II flow cytometer (Becton Dickinson, Franklin Lakes, NJ, USA). The data were analyzed against the isotype control using FlowJo software.

### 2.6. MMP Inhibitor GM6001 Preparation and Treatment

CHLA-02 and CHLA-05 cells were replated at a density of 1.6 × 10^6^ cells/mL. GM6001 (CC1000, Sigma Aldrich, St. Louis, MO, USA) was dissolved in DMSO to a stock concentration of 8 mM and added to the culture at a final concentration of 10 µM [62]. After 48 h, phase-contrast images of the aggregates were taken. Some cells were replated on *Matrigel*. In addition, the cells were harvested for RT-PCR analysis for epithelial, mesenchymal, and important ATRT biomarkers (MSLN, MUC16, and OPN).

### 2.7. Statistical Analysis

**Figure 1:**
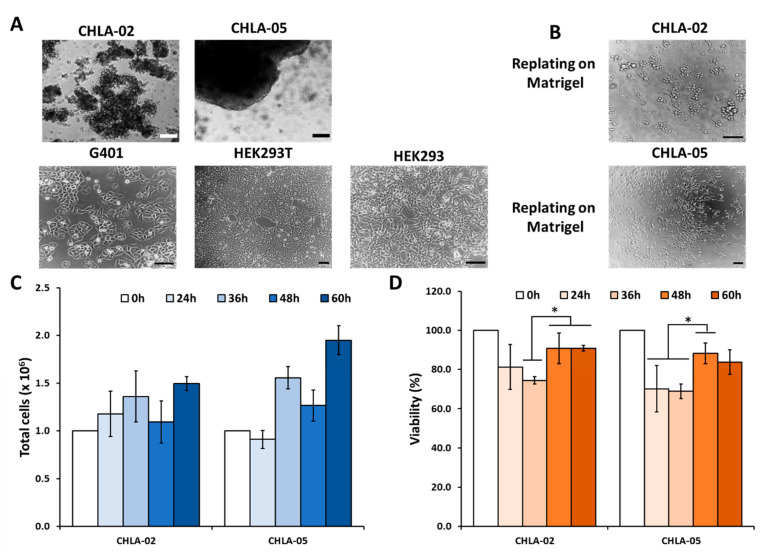
**Morphology, proliferation, and cell viability.** (**A**) The ATRT cell lines (CHLA-02 and CHLA-05) were suspended as aggregates when cultured in a normal well plate. The rhabdoid kidney tumor and the nonmalignant cell lines demonstrated attachment as a monolayer in culture. (**B**) Both ATRT cell lines showed attachment when cultured on a *Matrigel*-coated surface after 72 h. (**C**) Cell proliferation and (**D**) viability were measured at 24 h, 36 h, 48 h, and 60 h. *: *p*-value < 0.05, using the Student’s *t*-test. Scale bar: 200 µm.

**Figure 2:**
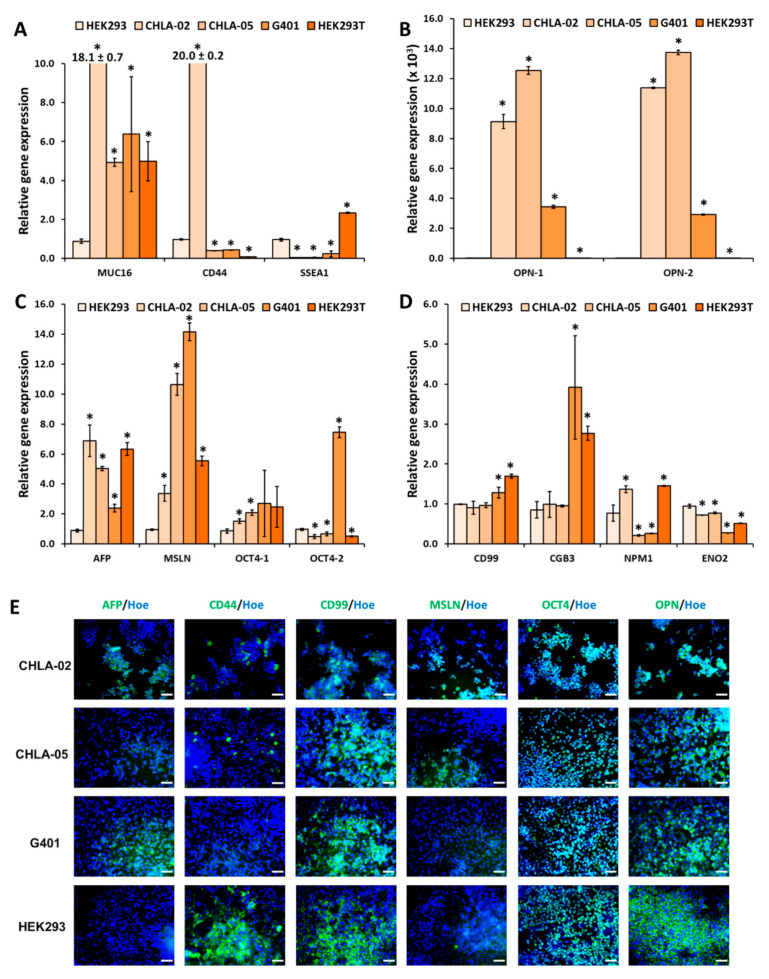
**Gene expression and immunofluorescence images of different cancerous biomarkers in rhabdoid tumors and nonmalignant cell lines.** Relative gene expression comparison of the nonmalignant cell line HEK293 with the ATRT cell lines CHLA-02 and CHLA-05, a kidney rhabdoid tumor cell line, G401, and the transformed HEK293T cell line, respectively. The markers of interest were: (**A**) *MUC16*, *CD44*, *SSEA1*; (**B**) *OPN*; (**C**) *AFP*, *MSLN*, *OCT4*; (**D**) *CD99*, *CGB3*, *NPM1*, and *ENO2*. For *OPN*, the relative gene expression levels for HEK293T and HEK293 were too low to be visible. *: *p*-value < 0.05 when compared with HEK293 using the Student’s *t*-test. (**E**) Immunostaining of several biomarkers of two ATRT cell lines, CHLA-02 and CHLA-05; a kidney rhabdoid tumor cell line, G401; and a nonmalignant cell line, HEK293. Scale bar: 100 μm.

**Figure 3:**
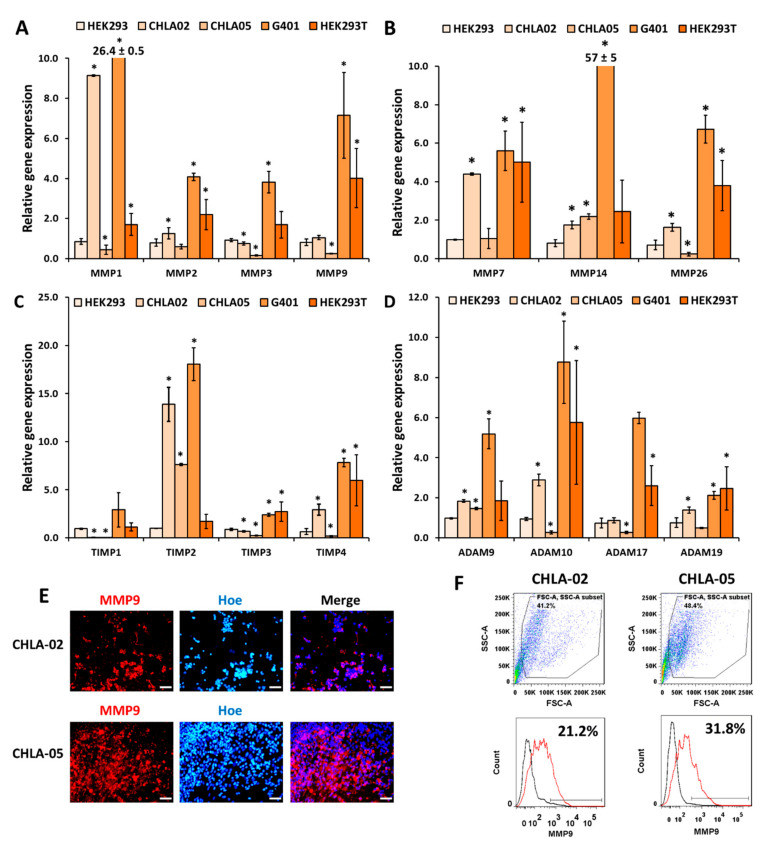
**Cellular expression of various MMPs, TIMPs, and ADAMs.** (**A**,**B**) Gene expressions of (**A**) *MMP1, 2, 3, 9*; (**B**) *MMP7, 14, 26*; (**C**) *TIMP1, 2, 3, 4*; (**D**) *ADAM9, 10, 17,* and *19*. *: *p*-value < 0.05 when compared to HEK293 cells using the Student’s *t*-test. (**E**) Immunostaining for MMP9. Scale bar: 100 μm. (**F**) Flow cytometry analysis of MMP9 expression in CHLA-02 and CHLA-05. Black line: isotype control; red line: the marker of interest.

**Figure 4:**
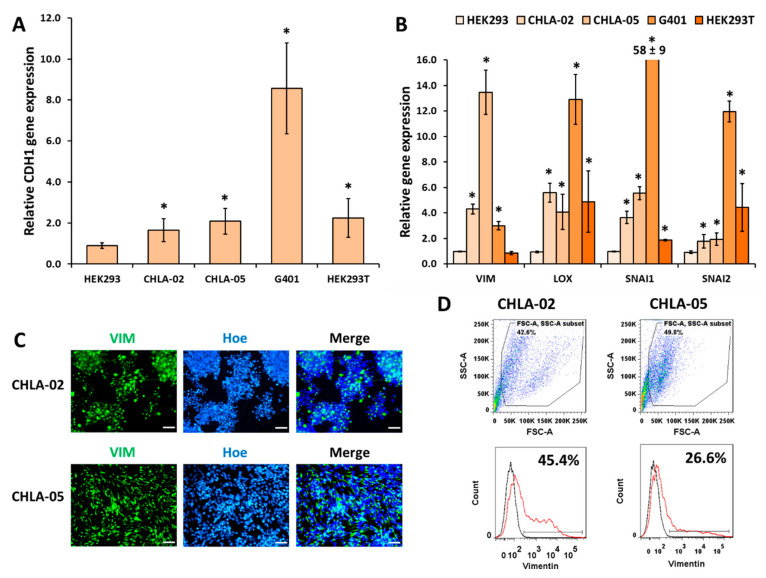
**Expression of epithelial and mesenchymal markers in the CHLA-ATRT and other cell lines.** Gene expressions for (**A**) the epithelial marker (*CDH1*) and (**B**) mesenchymal markers (*VIM*, *LOX*, *SNAI1*, and *SNAI2*). *: *p*-value < 0.05, when compared with HEK293 using the Student’s *t*-test. (**C**) Immunostaining for Vimentin (VIM). Scale bar: 100 μm. (**D**) The analysis of VIM expression by flow cytometry of both ATRT cell lines. Black line: isotype control; red line: markers of interest.

**Figure 5:**
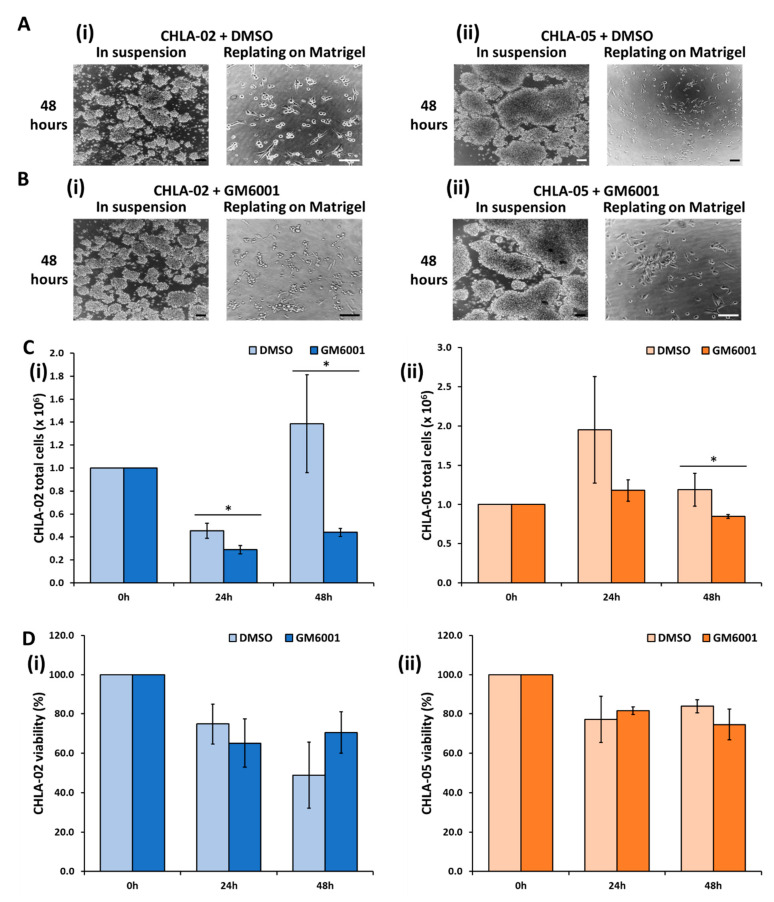
**The effect of GM6001 on the morphology, proliferation, and viability of the CHLA-02 and CHLA-05 cell lines.** The effect of (**A**) DMSO and (**B**) GM6001 treatment on (**i**) CHLA-02 and (**ii**) CHLA-05 morphology. Scale bar: 200 µm. (**C**) The cell proliferation and (**D**) viability of (**i**) CHLA-02 and (**ii**) CHLA-05 under the treatment of either DMSO or 10 µM of GM6001. *: *p*-value < 0.05, based on the Student’s *t*-test.

**Figure 6:**
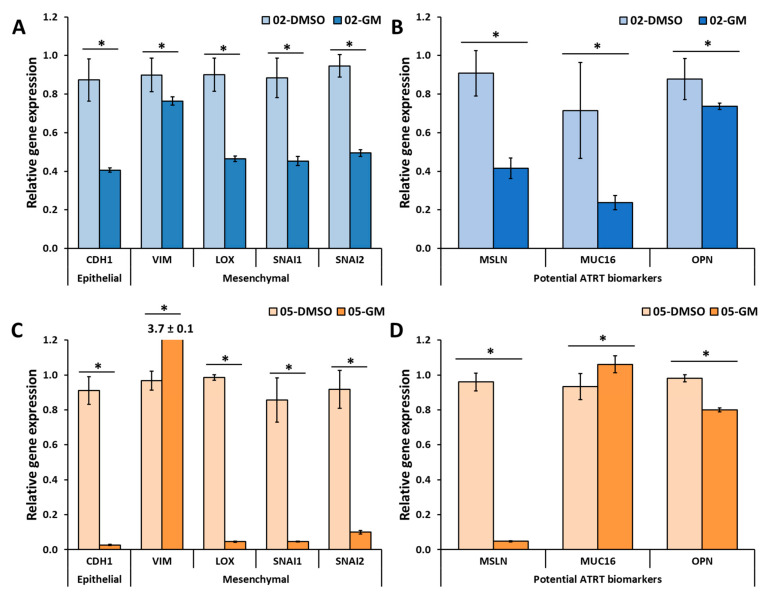
**The effect of GM6001 on the gene expression of CHLA-02 and CHLA-05.** (**A**,**B**) CHLA-02 and (**C**,**D**) CHLA-05 were treated with either DMSO or 10 µM GM6001, a broad-spectrum MMP inhibitor. The cells were characterized for various biomarkers. *The significant difference between the control (DMSO-treated) versus the GM6001 treatment was determined based on the Student’s *t*-test with a *p*-value < 0.05. * indicates statistically significant difference.

The RT-PCR, cell proliferation, and cell viability results were presented as means ± standard deviations. In addition, they were repeated as three independent data points (*n* = 3). To compare two conditions in Figure 1, Figure 2, Figure 3, Figure 4, Figure 5 and Figure 6, the Student’s *t*-test was used to determine the significance. A *p*-value < 0.05 was considered significant. On the other hand, for comparing more than two different conditions (Figure 1D), a one-way analysis of variance (ANOVA) was used, followed by Tukey’s post hoc test. Significance was determined with a *p*-value < 0.05.

## 3. Results

### 3.1. AFP, MSLN, OPN, and MUC16 Are Highly Expressed Biomarkers for ATRT

Unlike G401, HEK293T, and HEK293, both CHLA-02 and CHLA-05 formed aggregates in the long-term culture (Figure 1A); however, both ATRT cell lines could attach when replated on a *Matrigel*-coated surface. Some CHLA-02 cells retained their morphology similar to that in suspension culture; however, many CHLA-05 cells spread out entirely after 3 days of culture (Figure 1B). Both cell lines steadily increased their proliferation rates over time. At the time points of 24 h and 36 h, the CHLA-02 proliferation rate was similar to that of CHLA-05. After two days, the total population of CHLA-05 was higher than CHLA-02 (Figure 1C). In addition, CHLA-02 and CHLA-05 cells showed higher viability after 48 h of culture (Figure 1D).

The biomarkers (Table 1) were chosen based on published data for different cancer markers in the brain and in other common cancer types [23,28,63,64,65,66]. The gene expression of these markers was characterized using various primer pairs (Table S1). *MUC16*, *OPN*, *AFP*, and *MSLN* gene expression were highly expressed in all malignant rhabdoid tumor cell lines (CHLA-02, CHLA-05, and G401) (Figure 2A–D). The expression of *OPN* was extremely high for rhabdoid tumors, especially in both ATRT cell lines. On the other hand, *SSEA1* and *CD99* might not be good markers for ATRT. The low expression of *ENO2* for CHLA-02 and CHLA-05 suggests that these ATRT cells do not have neuronal characteristics in the brain. *CGB3* and *CD44* were not consistent among the ATRT samples. Specifically, *CGB3* was highly expressed in CHLA-05 but lower in CHLA-02 and in the nonmalignant cell line. *CD44* was highly expressed in CHLA-02 but low in CHLA-05. *OCT4* was inconclusive for ATRT, even though it is helpful as a marker for G401. *NPM1* has been proposed as a potential biomarker for ATRT [28], and the gene expression in both ATRT cell lines was lower than that in HEK293 (Figure 2A–D). The gene expression levels for all the primer pairs are presented in Figures S1 and S2 and Table S2.

Immunocytochemistry experiments were performed to detect the protein expression of several markers (Figure 2E, Tables S3 and S4, and Figures S3–S6). AFP was not expressed in HEK293; however, it was highly expressed in CHLA-02 and G401, though not in CHLA-05. CD44 was expressed in both ATRT cell lines and HEK293. CD99, OCT4, and OPN were well-expressed in all the rhabdoid tumor cell lines and HEK293. MSLN protein expression levels were low in both ATRT cell lines and in G401 and HEK293 cells, although the expression levels seemed to be slightly higher in CHLA-05, G401, and HEK293 cells.

### 3.2. CHLA-02 Cells Expresseed Higher Levels of MMPs Than CHLA-05 Cells

The gene expressions of several matrix metalloproteinases (*MMPs*), tissue inhibitors of metalloproteinases (*TIMPs*), and a disintegrin and metalloproteinases (*ADAMs*) were characterized for all five cell lines (Figure 3A–D, Tables S5 and S6). CHLA-05 cells had a lower expression of *MMP1*, *3*, *9*, *26*, *TIMP1*, *3*, *4*, *ADAM10*, and *17* than HEK293. CHLA-05 cells expressed a high MMP9 protein level (Figure 3E,F). CHLA-02 showed high gene expression levels of *MMP1*, *7*, *14*, and *26,* as well as a high MMP9 protein level (Figure 3). G401 cells expressed high levels of all the selected MMPs, especially *MMP14* (Figure 3A,B). These results indicate that CHLA-02 and G401 cells are highly invasive.

TIMP1 might not play an essential role in the cellular processes for ATRT cells due to its low expression in both CHLA-02 and CHLA-05; however, CHLA-02 expressed high levels of *TIMP2* and *4*, whereas CHLA-05 only showed an increased expression of *TIMP2*. *TIMP2*, *3*, and *4* were also expressed highly in G401 cells. HEK293T cells expressed a high level of *TIMP4* (Figure 3C). On the other hand, CHLA-02 cells showed high-level expression of the selected *ADAMs* but not *ADAM17*. Both CHLA-02 and CHLA-05 expressed *ADAM9*. G401 cells expressed all the studied *ADAMs* (Figure 3D), indicating that G401 cells participated highly in extracellular matrix remodeling.

### 3.3. Mesenchymal Markers Were Highly Upregulated in the Malignant Rhabdoid Tumors

All five cell lines were characterized for epithelial (*CDH1*) and mesenchymal markers (*VIM*, *LOX*, *SNAI1*, and *SNAI2*). The epithelial gene expression of *CDH1* was higher in all the cell lines when compared with HEK293 cells, and it was highest in G401 cells (Figure 4A). All the mesenchymal markers were highly expressed in both CHLA-05 and CHLA-02 (Figure 4B). Both ATRT cell lines expressed high levels of VIM based on ICC and flow cytometry (Figure 4C,D respectively).

### 3.4. GM6001 Significantly Decreased Cell Proliferation and the Gene Expression of Several Markers in Both ATRT Cell Lines

The effect of GM6001, a broad-spectrum MMPs inhibitor, on CHLA-02 and CHLA-05 was investigated. Under the treatment of 10 µM of GM6001, both cell lines did not show any differences in morphology in the aggregates or when replating them on a *Matrigel*-coated surface compared with the control (DMSO treatment) (Figure 5A,B). In addition, the treatment decreased cell proliferation compared with the control in both cell lines. The effect was more significant in the CHLA-02 than in the CHLA-05 and long-term cultures. In particular, GM6001 lowered the total cell number by 60% for CHLA-02 and by 30% for CHLA-05 (Figure 5C). Despite the lower cell proliferation, there were no significant differences in cell viability between the treated and untreated conditions (Figure 5D).

The effect of GM6001 on different biomarkers in both ATRT cell lines was also investigated using the RT-PCR method (Figure 6). The presence of GM6001 significantly decreased the gene expression by at least 50% of one epithelial marker (*CDH1*) and most mesenchymal markers (*LOX*, *SNAI1*, and *SNAI2*). The important ARTR biomarkers (*MSLN* and *OPN*) were also decreased by 16%. CHLA-05 showed an increase in the expression of *VIM* (~4 folds) and *MUC16* (~0.14 folds).

## 4. Discussion

The two human ATRT cell lines, CHLA-02 and CHLA-05, showed distinct differences in several gene expression markers. In many cases, the investigated MMP, TIMP, and ADAM gene expression levels in CHLA-02 were higher than in CHLA-05 (Tables S2 and S6), suggesting a highly invasive phenotype requiring more energy for the cellular proliferation of CHLA-02. CHLA-02 might also have more embryonic phenotypes than CHLA-05 due to high NPM1 gene expression and AFP positivity. This could be due to a high expression of MYC and high glutamine consumption in CHLA-02 [67]. In breast cancer patients, MYC expression increased the risk of breast cancer brain metastasis [68]. In addition, glutamine was used to fuel the TCA cycle, generate glutathione to counteract reactive oxygen species, and became a precursor for nucleic acid synthesis [69].

Among all the biomarkers investigated, OPN, AFP, MUC16, and MSLN may be the most important biomarkers for ATRT. OPN is a stem cell-promoting factor in glioblastoma [70], and its gene expression levels were high in all the rhabdoid tumor cell lines. OPN can activate CD44 by forming a protein complex that could lead to radiation treatment resistance in glioma [70]. The high expression level of *CD44* in CHLA-02 might suggest its invasive phenotype. AFP gene and protein expression may serve as a valuable biomarker for malignant rhabdoid tumors due to its much higher expression levels compared to nonmalignant cells.

MUC16 acts as a signaling molecule and a lubrication barrier of the epithelial surface [71]. The expression of MUC16 is associated with changes in signal transduction and gene expression that can lead to tumor invasion through the AKT/ERK pathway [72], similar to the function of the TIMP2–MMP14 complex [73,74]. Solid tumors that expressed MUC16 metastasized to MSLN-expressing locations, such as the peritoneum or pleura, in 75% of cases [75]. The ATRT cell lines expressed high levels of *MSLN* mRNA, but only low levels of protein were detected by immunocytochemistry (ICC), indicating that MSLN protein expression might be low, the proteins might be shed into the cell culture media, or that the sensitivity of the antibody is not sufficient. Therefore, targeting the ERK and AKT pathways and disrupting MSLN–MUC16 interactions may become novel strategies for ATRT treatment.

Our NPM1 and OCT4 expression results differed from those reported in the literature. Even though NMP1 has been suggested to be a potential target for ATRT treatment in the literature [28], *NMP1* gene expression was lower in both ARTR cell lines compared with the nonmalignant HEK293 cells in this study. This could be due to the different isoforms of NMP1. OCT4 is expressed in other cancer types, such as glioma, hepatocellular carcinoma, rectal cancer, and pancreatic cancer [76,77,78,79]. In this study, two primer pairs were used to detect the expression of *OCT4* mRNA in rhabdoid tumor cell lines, but the results were inconsistent with ATRT cell lines. Our results showed that gene expression levels were significantly higher in G401 than in HEK293 cells. At the protein level, all the rhabdoid tumor cell lines expressed OCT4, suggesting its important role in preserving the stem cell-like property. However, in other studies, the protein expression of OCT4 was not detected in ATRT or non-central nervous system malignant rhabdoid tumors [21,80,81,82,83]. Therefore, more studies should be carried out to investigate whether NPM1 and OCT4 would be useful biomarkers for ATRT in the future.

Investigating MMP and TIMP expression is important for understanding the invasiveness of ATRT cells. MMP1 is highly expressed in glioblastoma multiforme xenografts [84]. Furthermore, MMP1 induced the expression of proangiogenic genes in human microvascular endothelial cells by activating signaling MAPK cascades [85]. The high-level expression of MMP1 in both CHLA-02 and G401 may indicate a tumor microenvironment conducive to angiogenesis. In addition, CHLA-02 expressed a high level of *MMP-26*, an invasion biomarker in various cancers, including gliomas [86], astrocytic gliomas [87], colorectal cancer [88], and esophagus squamous cell carcinomas [89]. High MMP26 expression levels may indicate a poor prognosis in astrocytic glioma patients, and its expression is an independent factor associated with patient overall survival time [87]. MMP26 may promote cancer invasion and metastasis by activating proMMP-9 by cleaving the proenzyme site of Ala93-Met94 [89,90] and interacting with TIMP4 [91,92] to promote cancer progression. In addition, *MMP7* is a biomarker associated with the epithelial-to-mesenchymal transition (EMT) of tumor cells that are highly invasive [93,94], as well as colon cancer [95] and gliomas [96]. *MMP7* was highly expressed in CHLA-02. The gene expression profile of CHLA-02 demonstrated this cell line’s aggressive and invasive phenotype.

In addition to their inhibitory abilities against MMPs, TIMPs may promote cell proliferation and inhibit apoptosis [97]; however, brain malignant rhabdoid tumors do not express mRNA of *TIMP1* or *2* [98]. This study showed that the expression of *TIMP2* in both ATRT cell lines was significantly higher than in the nonmalignant HEK293 cell line. *TIMP4*, a biomarker for astrocytoma in glioma patients [99], was highly expressed in CHLA-02. In addition, TIMP-4 can induce tumor growth and promote tumor progenitor cells, possibly through the activation of the TGF-β and NF-κB protein network [100]. Lysyl Oxidase (*LOX*) and *SNAI2* were highly expressed in both ATRT cell lines. LOX and SNAI2 can regulate the induction of TIMP-4, which can lead to tumor invasion, migration, and VEGF expression [101].

*ADAM9* was highly expressed in both ATRT cell lines, but *ADAM10* was only expressed in CHLA-02. ADAM9 is an adhesive protein and can induce cell signaling, leading to increased proteolytic activities [102] and it is overexpressed in breast cancer and plays an important role in metastasis [103,104]. ADAM9 is related to poor tumor grade and cell proliferation, migration, and invasion [105,106,107], possibly due to mediation of the interaction between cancer cells and fibroblasts [102,108]. ADAM10 may play significant roles in neuronal functions and in the pathogenesis of Alzheimer’s disease [109,110]. It is overexpressed in glioblastoma and enhances cancer progression, migration, and immune evasion [111,112]. Moreover, re-expressing ADAM10 can lead to the restoration of a cell’s ability to proliferate, migrate, and invade; it is suppressed when overexpressing miR-365 [113,114].

Epithelial–mesenchymal transition (EMT) is a process in which cells change their phenotypes from polarized epithelial to mesenchymal [115,116]. This process is essential for embryogenesis, as well as cancer invasion and metastasis [115,116]. Both the ATRT cell lines and the kidney rhabdoid tumor cell line showed high-level gene expression of both epithelial and mesenchymal markers, suggesting that all three cell lines may have a hybrid phenotype with biomarkers that spread across the epithelial–mesenchymal spectrum. The ATRT cells form aggregates in suspension, but they also form attachments when cultured on *Matrigel*-coated surfaces, further supporting this hybrid phenotype hypothesis. Recently, two chromatin-modifying complexes, polycomb repressive complex 2 (PRC2) and lysine methyltransferase 2D-complex of proteins associated with SET1 (KMT2D-COMPASS), have been suggested to govern epithelial–mesenchymal plasticity [117]. Thus, ATRT and kidney malignant rhabdoid tumor cells have complex genotypes and phenotypes. In addition, cancer cell phenotypes could resemble the cancer stem cell phenotype during EMT, with increased drug efflux pumps and anti-apoptotic properties [118]. Therefore, small-molecule inhibitors or miRNA-targeting genes and pathways involved in EMT have been used to alleviate drug resistance [118].

GM6001 is a potent broad-spectrum MMP inhibitor. GM6001 treatment decreased the cell proliferation and gene expression of several important biomarkers in both CHLA-02 and CHLA-05, including MSLN, OPN, and many mesenchymal markers. The effect of GM6001 was more significant in CHLA-02, perhaps due to the higher MYC and MMP expression levels in CHLA-02 than in CHLA-05 [67]. In addition, there was an increase in *VIM* expression in CHLA-05. The suppression of MYC protein expression in colorectal cells by knocking down MMP1 [119] may suggest possible interactions between MYC and the MMPs. Therefore, the specific MMPs and their mechanisms for reducing cell proliferation and certain biomarkers, possibly by decreasing MYC expression, remain to be investigated.

Human embryonic kidney cell line HEK293 cells were used as nonmalignant controls for G401, a kidney-derived malignant rhabdoid tumor; however, HEK293 may not be the ideal control for ATRT cell lines. The origin of ATRT is still under investigation, but it might be in neural progenitor cells [120]; however, obtaining, culturing, and maintaining human pediatric undifferentiated neural progenitor cells might be challenging and not feasible. Since both fetal non-pluripotent neural progenitor cells [121] and HEK293 cells express pluripotency markers of NANOG and OCT4 [122], HEK293 was used as a nonmalignant stem cell control for comparison in this study.

This comprehensive study investigated biomarkers of different types, epithelial, mesenchymal, MMPs, TIMPs, and ADAMs, as well as the effects of GM6001 on ATRT cells; however, these studies were limited to using two ATRT cell lines, one malignant kidney rhabdoid tumor cell line (G401), and two kidney control cell lines HEK293 and HEK293T. In the future, other ATRT cell lines and patient-derived xenograft samples need to be investigated to verify these ATRT antigens. Although CHLA-02 and CHLA-05 have different cellular pathways and metabolic regulations, they might not be able to represent all ATRT patient-derived cell lines and tissues. Thus, future large-scale studies are needed to verify our findings for therapeutic development to treat patients with ATRT and other malignant rhabdoid tumors.

## 5. Conclusions

In summary, CHLA-02 and CHLA-05 may represent two different subtypes of ATRT based on their gene expression profiles. AFP, MSLN, MUC16, OPN, and MT1-MMP/MMP14 may serve as biomarkers for human malignant rhabdoid tumor detection and therapeutic targets. Due to their high MT1-MMP and other MMP expression levels, ATRT cells may be highly invasive. Treating ATRT with a broad-spectrum MMP inhibitor, GM6001, decreased cell proliferation with no significant changes in viability or several important markers in both ATRT cell lines. ATRT may utilize the MYC and ERK pathways as their central metabolic regulation, suggesting an important therapeutic intervention approach. OPN and MSLN may serve as targets for immunotherapies and cancer vaccine development to treat ATRT patients. This study may also advance our knowledge of the molecular features of malignant rhabdoid tumors and their aggressiveness.

## Figures and Tables

**Table 1:** Biomarkers investigated in this study.

Type	Gene	Name	Location
**ATRT**	**OPN**	Osteopontin	Secreted
	**NPM1**	Nucleophosmin 1	Intracellular
**Common** **cancer** **biomarkers**	**MUC16**	Mucin-16	Membrane, secreted
	**CD44**	Homing cell adhesion molecule	Intracellular, membrane, secreted
	**AFP**	Alpha-fetoprotein	Secreted
	**MSLN**	Mesothelin	Intracellular, membrane
	**CD99**	Single-chain type-1 glycoprotein	Intracellular, membrane
	**CGB3**	Chorionic gonadotropin β-3	Secreted
**Embryonic**	**SSEA1**	Fucosyltransferase 4	Membrane
	**OCT4**	Octamer-binding transcription factor 4	Intracellular
**Neuronal**	**ENO2**	Neuron-specific enolase	Intracellular

## Data Availability

The datasets generated and used/or analyzed are published in this paper and Supplementary Materials, and available from the corresponding author upon request.

## Supplementary Materials

The following supporting information can be downloaded at: https://www.mdpi.com/article/10.3390/cancers14153685/s1, Figure S1: The relative gene expression of (A) MUC16, (B) CD44, (C). SSEA1, (D) OPN, (E) AFP, and (F) MSLN using different primer pairs from Table S1. The gene expression levels of CHLA-02, CHLA-05, G401, and HEK293T cells were compared with HEK293 using the Student’s *t*-test. *: *p*-value < 0.05, Figure S2: Relative gene expression levels of (A) OCT4, (B) CD99, (C) CGB3, (D) NPM1, and (E) ENO2 using different primer pairs from Table S1. The gene expression levels of CHLA-02, CHLA-05, G401, and HEK293T cells were compared with HEK293 using the Student’s *t*-test. *: *p*-value < 0.05, Figure S3: Immunostaining of different cancerous biomarkers for CHLA-02. Scale bar: 100 µm, Figure S4: Immunostaining of different cancerous biomarkers for CHLA-05. Scale bar: 100 µm, Figure S5: Immunostaining of different cancerous biomarkers for G401. Scale bar: 100 µm, Figure S6: Immunostaining of different cancerous biomarkers for HEK293. Scale bar: 100 µm, Table S1: Primer sequences for the markers of interest, Table S2: Comparison of the relative gene expression levels of the tested biomarkers of CHLA-02, CHLA-05, G401, HEK293T with those of HEK293, Table S3: List of primary antibodies, Table S4: List of secondary antibodies, Table S5: The primer sequences for the MMPs, TIMPs, and ADAMs, Table S6: Comparison of the relative gene expression levels of extracellular matrix modifier markers of CHLA-02, CHLA-05, G401, HEK293T with those of HEK293, Table S7: The primer sequences for the epithelial (CDH1) and mesenchymal markers (LOX, SNAI1, SNAI2, and VIM).
