# Supplementary material for: Human Malignant Rhabdoid Tumor Antigens as Biomarkers and Potential Therapeutic Targets
Source: Cancers (Basel). 2022 Jul 28;14(15):3685. doi: 10.3390/cancers14153685 (PMC9367328; doi:10.3390/cancers14153685)
Supplement: Supplementary file 1 [file cancers-14-03685-s001.zip › cancers-1806748-supplementary.pdf]

cancers MDPI

Supplementary Materials

Human Malignant Rhabdoid Tumor Antigens as Biomarkers and Potential Therapeutic Targets

Timothy Hua [1], Ziwei Zeng [1], Junji Chen [1], Yu Xue [1], Yan Li [2,3] and Qing-Xiang Sang [1,3,*]

1 Department of Chemistry and Biochemistry, Florida State University, Tallahassee, FL 32306-4390, USA; tph16c@fsu.edu (T.H.); zengzw@mail2.sysu.edu.cn (Z.Z.); chenjj47@mail2.sysu.edu.cn (J.C.); yx21@fsu.edu (Y.X.)
2 Department of Chemical and Biomedical Engineering, FAMU-FSU College of Engineering, Florida State University, Tallahassee, FL 32310-6046, USA; yli4@fsu.edu
3 Institute of Molecular Biophysics, Florida State University, Tallahassee, FL 32306-4380, USA
* Correspondence: qxsang@chem.fsu.edu; Tel.: 1-850-644-8683; Fax: 1-850-644-8281

Table S1. The primer sequences for the markers of interest.

Gene	Forward sequence (5′-3′)	Reversed sequence (5′-3′)
AFP	CCCTCCTGCATTCTCTGATGA	TGGCTTTTGCTTCACAAGGTT
	TGTAGCGCTGCAAACAATGAA	ACAGGCCTGAGAAATCTGCAA
	CTGCTTTGCTGAAGAGGGACA	TTCACACCGAATGAAAGACTCG
MSLN	GAAGTCAACAAAGGGCACGAA	CTTTGTCTAGCTGGCCCCTTC
	AGAACATGAACGGGTCCGAAT	CCATGCTCACATTCTGCTGAC
	TCAGTCAGCAGAATGTGAGCA	CCAGAAGTTTCTGCACCTCAGC
OCT4	ACCCACACTGCAGCAGATCA	GTTGTGCATAGTCGCTGCTTG
	CCCATGCATTCAAACTGAGGT	AAAAACCCTGGCACAAACTCC
OPN	ACAGCCACAAGCAGTCCAGAT	GAATTCACGGCTGACTTTGGA
	AGCGGAAAGCCAATGATGAGAGC	ACTTTTGGGGTCTACAACCAGCAT
MUC16	TGAGGAATTTCTGCGGATGAC	TGTTGGGAGAATACCCATCCA
	CCAGGACAAGGTCACCACACT	TGCCTTGACAGTGACCAACAC
	GGGAGTCATCACATGCCTGAT	TAGTAGCCTGGGCACTGTTGC
	ACATCCAGGACAAGGTCACCA	ACACGGAGTCCATCGTCAAGT
CD44	TGCCTCTTGTTTTCCCAGAGA	TTGAACCTTGTCCTGGAGTGG
	AGCTGGCCAAGTCTTCACAAA	TCCAGAGTTACGCCCTTGAGA
SSEA1	GCAAACGTTTTTCCAGGGAGTA	ACATGCCAGGCACTATCCTGA
	GCATGTAGGAAGCACCTGGAA	CTGGTTCTGCCACTGCTATTG
	TGTTGGGACCTCCTAGTTCCA	TCCAGACTGTAAGGAAGCCACA
CD99	TTGGCAGCAGGGTTAGAACAG	AAGCAAAGCACATCGCAAGAT
	CCCTTCCTGGGGATGACTTT	AACTAGGGTGGTTGGGGTTTG
CGB3	TCCAGGACTCCTCTTCCTCAAA	GCCTTTATTGTGGGAGGATCG
	CTCACCCCAGCATCCTATCAC	CTGGAACATCTCCATCCTTGGT
ENO2	TATAAAAGGGGGTCCGTGGAA	ACACGTGGGACAAGAGCAAAG
	ACTTGGGGGAACGATGTGTCT	TGTCAAGGCCCTTTCTATGACC
NPM1	TGTTCTCTGGAGCAGCGTTCT	CATGTCCATGTCCATCGAATCT
	CGTCCTTTCCCTGGTGTGATT	GGCACGCACTTAGGTAGGAGA

Table S2. The comparison of the relative gene expression of the tested biomarkers of CHLA-02, CHLA-05, G401, HEK293T to HEK293.

Type	Gene	CHLA02	CHLA05	G401	HEK293T
ATRT	OPN	Higher	Higher	Higher	Lower
	NPM1	Lower	Higher	Higher	Lower
Common	MUC16	Higher	Higher	Higher	Higher
	CD44	Higher	Lower	#	Lower
	AFP	Higher	Higher	Higher	Higher
	MSLN	Higher*	Higher	Higher	Higher
	CD99	Not significantly different	Not significantly different	Higher	Higher
	CGB3	Lower*	Higher	Higher	#
Embryonic	SSEA1	Lower	Lower	Lower	Higher
	OCT4	#	#	Higher**	#
Neuronal	ENO2	Lower	Lower	Lower	Lower

#: The results were inconsistent. Therefore, these markers require more investigation for these cell lines. *: For CHLA-02, the average MSLN relative gene expression average was higher in all the tested primers. However, only primer pair 1 yielded a significantly higher MSLN relative gene expression than HEK293. In addition, CGB3 expression was only significantly lower in primer pair 2. **: G401 showed a higher average OCT4 gene expression, but only primer pair 2 showed a significantly higher expression than HEK293.

Table S3. The list of primary antibodies.

**Protein name**	**Product name**	**Vendor**	**Cat. #**
OCT-4	Anti-OCT-4 [POU5F1]	Millipore	MAB4419
α-fetoprotein	AFP Antibody (39)	Santa Cruz	sc-130302
Vimentin	Vimentin (3CB2)	Santa Cruz	sc-80975
CD44	H4C4	DSHB	H4C4
CD99	MIC2 Antibody (12E7)	Santa Cruz	sc-53148
Mesothelin	Mesothelin Antibody (G-1)	Santa Cruz	sc-271540
Osteopontin	MPIIIB10(1)	DSHB	MPIIIB10(1)
MMP9	MMP-9 Antibody (C-20)	Santa Cruz	sc-6840

Table S4. The list of secondary antibodies.

**Product name**	**Vendor**	**Cat.** #
Alexa Fluor 488, goat anti-mouse IgM	Thermo Fisher Scientific	A-21042
Alexa Fluor 594, goat anti-rabbit IgG(H+L)	Thermo Fisher Scientific	A-11012

Table S5. The primer sequences for the MMPs, TIMPs, and ADAMs.

Gene	Forward	Reversed
**MMP1**	GGGAGATCATCGGGACAACTC	AAAATGAGCATCCCCTCCAAT
**MMP2**	ACTGTCTCAAGAGGGCACTGGT	AGGTTCTAAGGCAGCCAGCAG
**MMP3**	GTTCGTTTTCTCCTGCCTGTG	AAGCTAAGCAGCAGCCCATTT
**MMP7**	TGGAAATGGAGATCCCCAAAA	TGAATGGATGTTCTGCCTGAAG
**MMP9**	CGCTGGGCTTAGATCATTCCT	CCATTCACGTCGTCCTTATGC
**MMP14**	GGGTCTTCGTTGCTCAGTCAG	CACTTAGTCCCCAGTGGCTCA
**MMP26**	TGGGACTTTGTTGAGGGCTAT	TTGTTGCAGGAGCTGTGTTTG
**TIMP1**	AGTTTTGTGGCTCCCTGGAA	TTGCAGGGGATGGATAAACAG
**TIMP2**	CATTTGACCCAGAGTGGAACG	GGAGAACCAAAGACGGGAGAC
**TIMP3**	ATCAAGGAAGCTCCATGCTCA	CGAGGGGAGGGTAAAAGACAG
**TIMP4**	CCTAACGAGTGCCTCTGGACA	GTGCCGTCAACATGCTTCATA
**ADAM9**	CAAAAGAATGCACAAGAACCACA	GGAAGCGAGTAACTGCGTGAA
**ADAM10**	TGGCTACTTCAGCTCCCATTC	TTCCTTCCGCTAGACCCTCAG
**ADAM17**	TTTTGTGAAGACTGGGAAGTGAC	TCAAAAGGAGAAGGGCCAAAC
**ADAM19**	TCTGCTGGACACCCAAGAAAG	GGAAATGTGGGCTTGGATTCT

Table S6. The comparison of the relative gene expression of extracellular matrix modifier markers of CHLA-02, CHLA-05, G401, HEK293T to HEK293.

Markers	CHLA02	CHLA05	G401	HEK293T
MMP1	Higher	Lower	Higher	Higher
MMP2	Higher	Not significantly different	Higher	Higher
MMP3	Lower	Lower	Higher	Not significantly different
MMP7	Higher	Not significantly different	Higher	Higher
MMP9	Not significantly different	Lower	Higher	Higher
MMP14	Higher	Higher	Higher	Higher
MMP26	Higher	Lower	Higher	Higher
TIMP1	Lower	Lower	Higher	Not significantly different
TIMP2	Higher	Higher	Higher	Higher
TIMP3	Lower	Lower	Higher	Higher
TIMP4	Higher	Lower	Higher	Higher
ADAM9	Higher	Higher	Higher	Higher
ADAM10	Higher	Lower	Higher	Higher
ADAM17	Not significantly different	Lower	Higher	Higher
ADAM19	Higher	Not significantly different	Higher	Higher

Table S7. The primer sequences for the epithelial (CDH1) and mesenchymal markers (LOX, SNAI1, SNAI2, and VIM).

Gene	Forward	Reversed
**CDH1**	AACACATTTGCCCAATTCCAG	ACCCCTCAACTAACCCCCTTT
**VIM**	TGGAAGAGAACTTTGCCGTTG	ACGAGCCATTTCCTCCTTCAT
**SNAI1**	CCCTCCACGAGGTGTGACTAA	CTCGGGGCATCTCAGACTCTA
**SNAI2**	ATGAGGAATCTGGCTGCTGTG	ATTTGTCATTTGGCTTCGGAGT
**LOX**	CCTGGCACCTCTAAACACACC	TGAGTTTCGGCAGTTATTCAGAGA

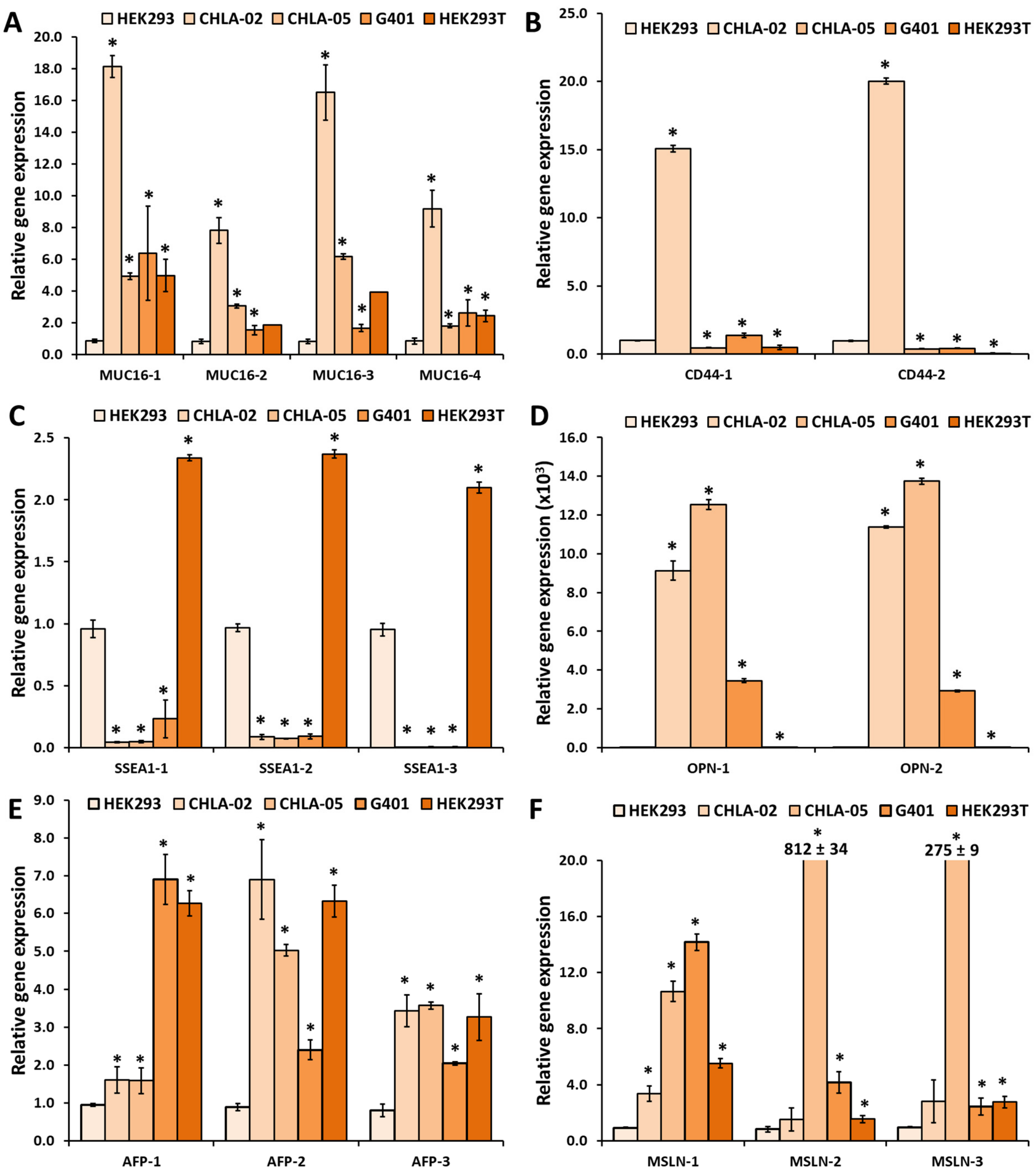

Figure S1. The relative gene expression of (A) MUC16, (B) CD44, (C) SSEA1, (D) OPN, (E) AFP, and (F) MSLN using different primer pairs from the Supplementary Table 1. The gene expression levels of CHLA-02, CHLA-05, G401, and HEK293T cells were compared with HEK293 using Student's t-test. *: p-value < 0.05.

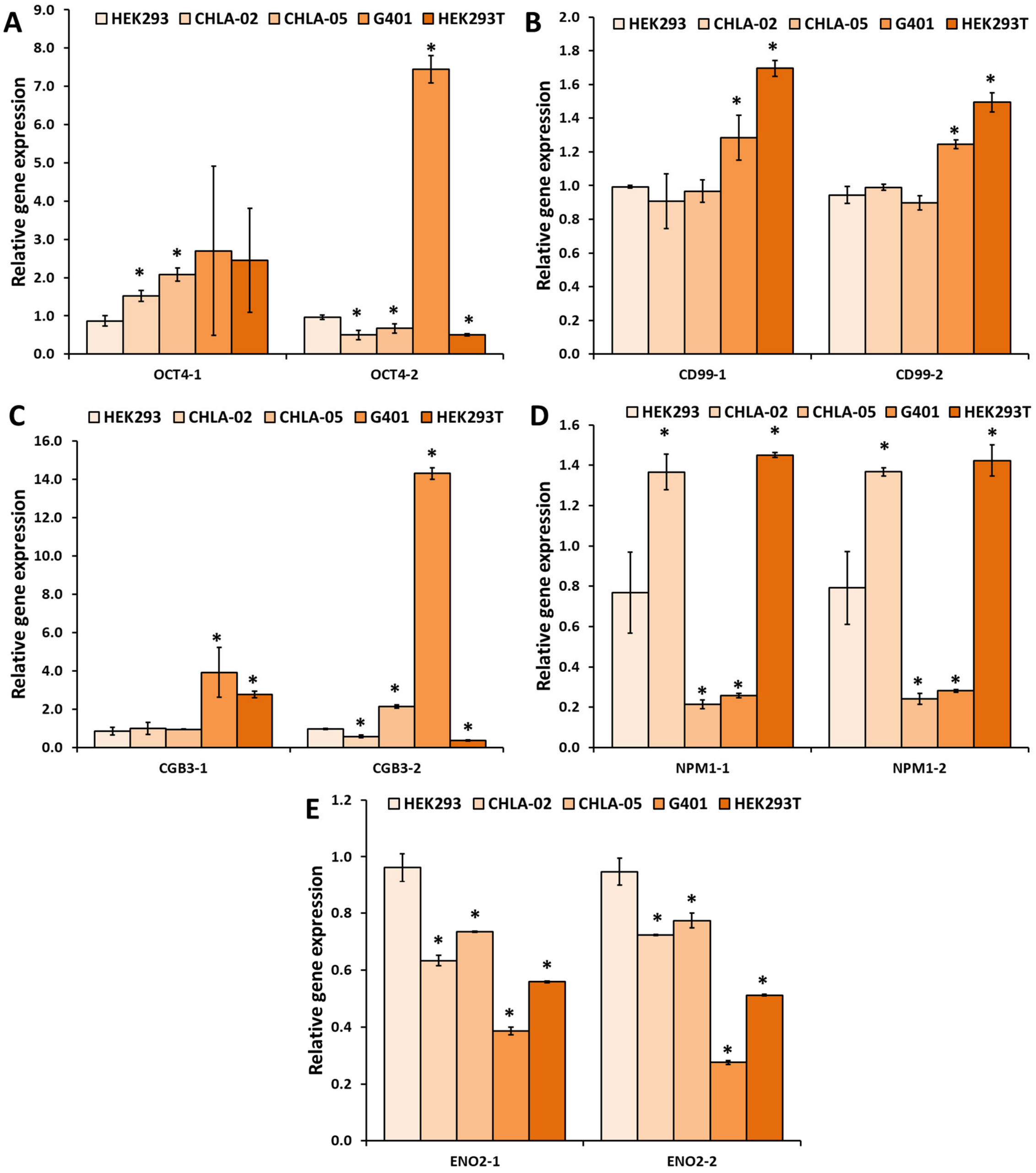

Figure S2. The relative gene expression of (A) OCT4, (B) CD99, (C) CGB3, (D) NPM1, and (E) ENO2 using different primer pairs from the Supplementary Table 1. The gene expression levels of CHLA-02, CHLA-05, G401, and HEK293T cells were compared with HEK293 using Student's t-test. *: *p*-value < 0.05.

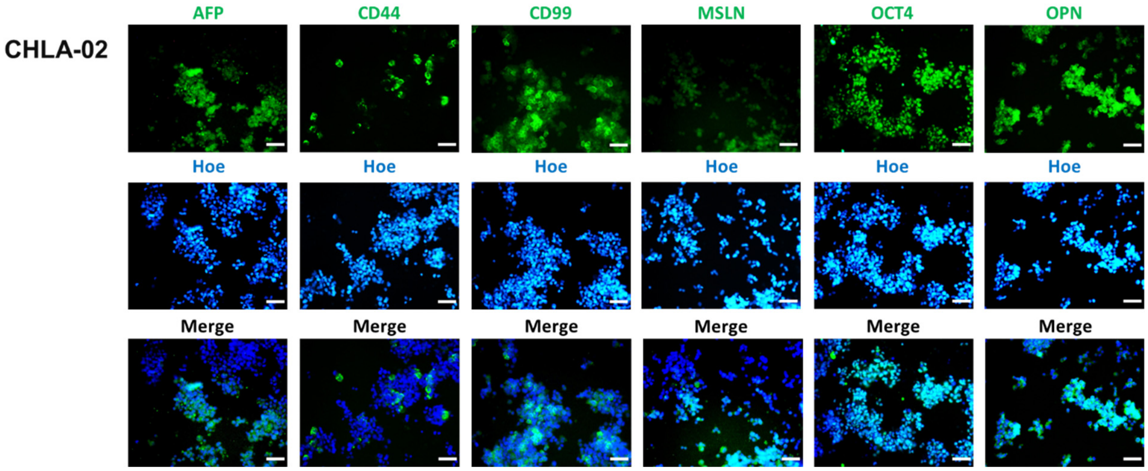

Figure S3. The immunostaining of different cancerous biomarkers for CHLA-02. Scale bar: 100 μm.

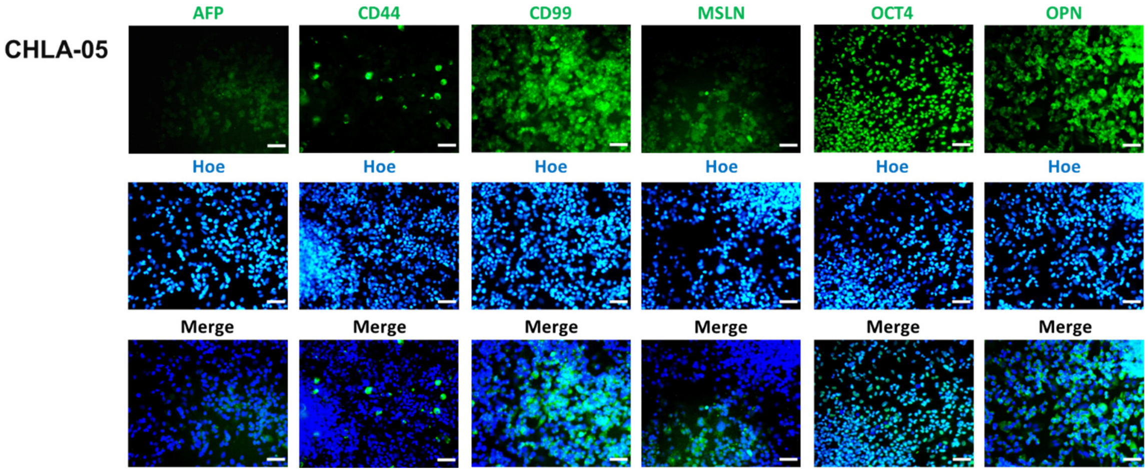

Figure S4. The immunostaining of different cancerous biomarkers for CHLA-05. Scale bar: 100 μm.

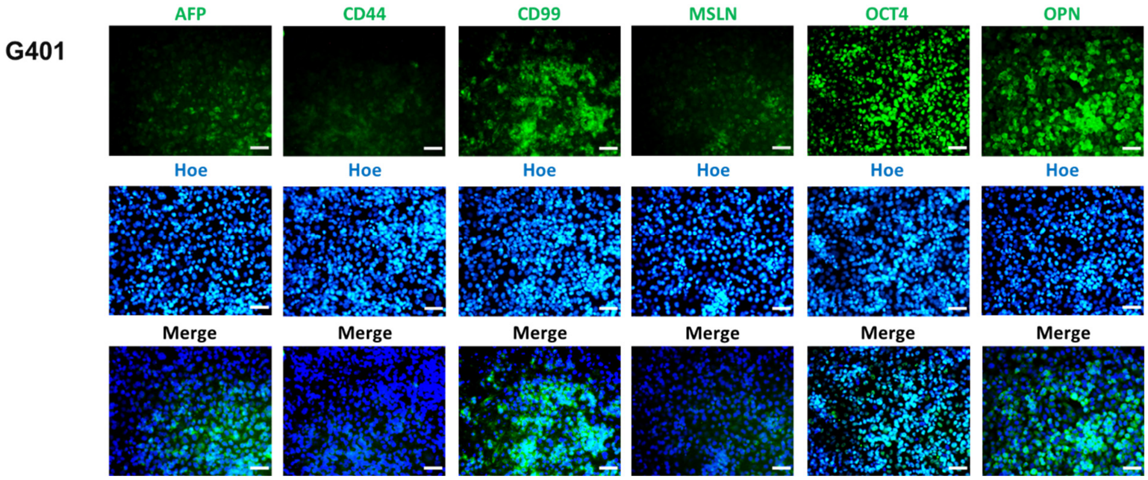

Figure S5. The immunostaining of different cancerous biomarkers for G401. Scale bar: 100 μm.

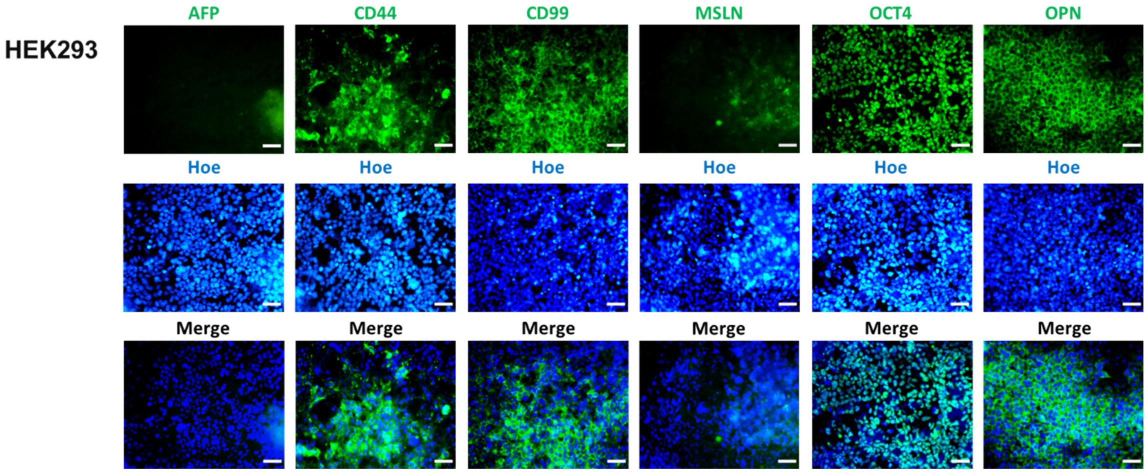

Figure S6. The immunostaining of different cancerous biomarkers for HEK293. Scale bar: 100 μm.
